# CRISPR-Cas12a/RPA Dual-Readout Assay for Rapid Field Detection of Porcine Rotavirus with Visualization

**DOI:** 10.3390/v17070872

**Published:** 2025-06-20

**Authors:** Xinjie Jiang, Yun Huang, Yi Jiang, Guang Yang, Xiaocong Zheng, Shuai Gao

**Affiliations:** 1 Hainan Laboratory Animal Research Center, Sanya Institute of Hainan Academy of Agricultural Sciences, Sanya 572000, China; jiangxinjie2025@163.com (X.J.); xiaoyistudent@foxmail.com (Y.J.); 2 Animal and Plant Inspection and Quarantine Technology Center of Shenzhen Customs, Shenzhen 518045, China; 69057285hy@sina.com; 3 College of Animal Science and Technology, Sanya Institute of China Agricultural University, Sanya 572025, China; yangguangimu@163.com

**Keywords:** porcine rotavirus, CRISPR/Cas12a, RPA, visual detection, field-deployable diagnostics

## Abstract

PoRV is a significant etiological agent of neonatal diarrhea in piglets, resulting in substantial economic losses within the global swine industry due to elevated mortality rates and reduced productivity. To address the urgent need for accessible and rapid diagnostics in resource-limited settings, we have developed a CRISPR/Cas12a-based assay integrated with recombinase polymerase amplification (RPA) for the visual detection of PoRV. This platform specifically targets the conserved VP6 gene using optimized RPA primers and crRNA, harnessing Cas12a’s collateral cleavage activity to enable dual-readout via fluorescence or lateral flow dipsticks (LFDs). The assay demonstrates a detection limit of 10^2^ copies/μL within 1 h, exhibiting no cross-reactivity with phylogenetically related pathogens such as Transmissible Gastroenteritis Virus (TGEV). By eliminating reliance on thermal cyclers or specialized equipment, this method is fully deployable in swine farms, veterinary clinics, or field environments. The lateral flow format provides immediate colorimetric results that require minimal technical expertise, while the fluorescence mode allows for semi-quantitative analysis. This study presents a robust and cost-effective platform for decentralized PoRV surveillance in swine populations, addressing the critical need for portable diagnostics in resource-limited settings and enhancing veterinary health management.

## 1. Introduction

Porcine rotavirus (PoRV) poses significant economic challenges to the global swine industry, primarily characterized by high morbidity in piglets, severe diarrhea, dehydration, and growth retardation, leading to increased mortality and reduced production performance [1,2]. The virus frequently co-infects with other pathogens such as Porcine Epidemic Diarrhea Virus (PEDV), exacerbating diagnostic and control complexities while elevating treatment costs [3]. Continuous viral evolution has limited the cross-protective efficacy of existing vaccines, underscoring the critical need to develop sensitive and specific detection methods for timely disease surveillance, precise pathogen differentiation, and targeted prevention strategies [4]. Expedited diagnosis technologies, including real-time fluorescent reverse transcription recombinase-aided amplification (RT-RAA) and multiplex qRT-PCR, enable large-scale screening and early diagnosis, thereby effectively curbing epidemic spread and minimizing economic losses. Consequently, enhancing PoRV detection capabilities represents a critical measure to control transmission and mitigate financial burdens in swine farming [5,6,7].

However, current diagnostic assays for PoRV exhibit multiple limitations [8]. Firstly, conventional diagnostic approaches, such as polyacrylamide gel electrophoresis (PAGE) and reverse transcription polymerase chain reaction (RT-PCR), are laboratory-dependent, unsuitable for on-site rapid detection, and involve complex procedures with prolonged processing times [9]. Secondly, existing methods lack sufficient sensitivity, specificity, and reproducibility, particularly in cases of co-infections (e.g., with PEDV), impairing the precise differentiation of pathogens [10,11]. Furthermore, while molecular techniques like RT-PCR can detect viral nucleic acids, they cannot evaluate viral infectivity [12]. Viral culture techniques, though valuable for viability analysis, present inherent technical challenges and limited practicality. Age-dependent discrepancies in detection efficacy also persist; for instance, low PoRV detection rates in suckling piglets may correlate with suboptimal sampling intervals or the inadequate sensitivity of current methods [13]. These constraints collectively underscore the urgent need to develop rapid, highly sensitive, and field-deployable integrated detection technologies.

The development trends of CRISPR-based diagnostic technologies for viral detection are primarily reflected in the following aspects [14]: First, CRISPR-Cas systems (e.g., Cas12, Cas13) have become core tools for viral detection due to their high sensitivity and specificity, enabling the rapid identification of DNA/RNA viruses, including highly variable strains such as Crimean–Congo Hemorrhagic Fever Virus (CCHFV) [15]. Second, the technology is advancing toward portability and point-of-care testing, exemplified by SHERLOCK and DETECTR systems, which achieve equipment-free on-site testing [16]. Third, multiplex detection capabilities have been enhanced through the design of multiple gRNAs or the integration with microfluidic technologies, allowing the simultaneous detection of multiple pathogens or viral variants. Fourth, the detection scope has expanded from nucleic acids to non-nucleic acid targets, including viral proteins, drug-resistant mutations, and cancer-related viral biomarkers [17]. Additionally, technological optimizations have significantly reduced costs (by 90% compared to traditional PCR) and shortened processing times to within 30 min. However, challenges such as gRNA design efficiency and false-positive control remain. Future breakthroughs are expected through AI-assisted gRNA screening, the development of novel Cas enzymes (e.g., Cas14), and integration with other technologies (e.g., NGS and nanomaterials). These advancements will establish CRISPR diagnostics as a transformative tool for addressing emerging infectious diseases and viral mutations [11,18,19,20].

## 2. Materials and Methods

The CRISPR-Cas-mediated detection of PoRV involves three core steps: (1) Isothermal amplification of viral RNA via RPA, converting trace RNA to detectable levels; (2) CRISPR-Cas12a activation, where amplified DNA triggers Cas12a’s collateral cleavage activity; and (3) fluorescent signal generation via the cleavage of reporter probes, enabling real-time visualization. This workflow leverages Cas12a’s high specificity for PoRV genomic sequences and RPA’s rapid amplification at a constant temperature (37 °C), eliminating the need for thermocycling.

### 2.1. Plasmid, Virus Strain, and Genome

Transmissible Gastroenteritis Virus (TGEV) was provided and maintained by the Lanzhou Veterinary Research Institute, Chinese Academy of Agricultural Sciences. The Porcine Rotavirus Nucleic Acid Quality Control (QC) Material (Lot No. 20240501) was manufactured by Xinyang Laiyao Biotechnology Co., Ltd. (Xinyang, China).

### 2.2. RNA Extraction

Viral RNA from TGEV and PoRV was extracted using the TaKaRa MiniBEST Viral RNA/DNA Extraction Kit (TaKaRa Bio Inc., Kyoto, Japan), following the manufacturer’s protocol. RNA concentration and purity were measured using a NanoDrop 2000 spectrophotometer (Thermo Fisher Scientific, Waltham, MA, USA).

### 2.3. Recombinase Polymerase Amplification (RPA) Primer Screening

The plasmid standard pUC57-PoRV VP6 (The plasmids were self-prepared in the laboratory, not commercially available standard plasmids)was serially diluted in 10-fold gradients to generate concentrations ranging from 1.16 × 10^5^ to 1.16 × 10^0^ copies/μL. Primer pairs (F1/R1, F2/R2, F3/R3, F3/R4, F4/R3, F4/R4, F5/R5, F5/R6, F6/R5, F7/R6) were systematically evaluated using these diluted standards (Table 1).

The RT-RPA reaction system was prepared according to the Basic RT-RPA Nucleic Acid Amplification Kit (Zhongce Biotechnology Co., Ltd., Hangzhou, China). Each 20 μL reaction contained 0.8 μL each of the forward and reverse primers (10 μM), 4.64 μL diethylpyrocarbonate (DEPC)-treated water, 11.76 μL Buffer A, and lyophilized enzyme pellets pre-aliquoted in 0.2 mL tubes. After adding 1 μL template DNA and 1 μL MgOAc (280 mM), the reactions were vortexed, briefly centrifuged to collect liquid, and incubated at 39 °C for 30 min. Amplification products were analyzed via 1% agarose gel electrophoresis using a Gel Doc™ XR+ Imaging System (Bio-Rad Laboratories, Hercules, CA, USA), with the optimal primer pair selected for subsequent experiments.

### 2.4. Design and Screening of crRNA Primers

The VP6 gene fragment of porcine rotavirus (GenBank accession: KU886317.1) was selected as the detection target. Approximately 200 full-length VP6 gene sequences of PoRV were retrieved from the NCBI database and aligned using MEGA 7.0.26 software (Mega Limited, Auckland, New Zealand) to identify conserved regions. Nine crRNAs targeting the PAM (Protospacer Adjacent Motif) sequences of the VP6 gene were designed, incorporating a reverse-complementary sequence followed by the LbCas12a protein-specific hairpin structure (Table 2).

crRNA synthesis was performed using the T7 High Efficiency In Vitro Transcription Kit (Sangon Biotech, Shanghai, China), followed by purification with the Centrifugal Column RNA Cleanup Kit (Sangon Biotech). All primers and probes were synthesized by Sangon Biotech.

For crRNA screening, RNA extracted from PoRV-positive samples was reverse-transcribed and amplified using primers F1/R1, F3/R4, F4/R4, F5/R5, F6/R5, and F7/R6 (Table 1). The CRISPR/Cas12a reaction system contained 1 μL LbCas12a (1 μM, NEB #M0653T), 3 μL 10× Cas12a Reaction Buffer, 1.2 μL ssDNA reporter (10 μM), 1 μL PCR product, and 1 μL individual crRNA (10 μM) in a total volume of 22.8 μL with DEPC-treated water. The reactions were incubated at 39 °C for 30 min using a TwistDx T16 portable isothermal fluorescence detector (TwistDx Inc., Maidenhead, UK). Each crRNA was tested in triplicate.

The optimal crRNA demonstrating the maximal fluorescence signal-to-noise ratio was selected for subsequent experiments (Table 2). This crRNA was then integrated into the RAA reaction system for PoRV detection.

### 2.5. Optimization of RT-RAA Product Volume

To determine the optimal input volume for CRISPR/Cas12a detection, RT-RAA amplification products (generated with primers PoRV-RAA-F2/R2 as described in Section 2.5) were tested at volumes of 10 μL, 8 μL, 6 μL, 4 μL, 2 μL, and 1 μL in the CRISPR/Cas12a reaction. The reaction system comprised 1 μL LbCas12a (0.1 μM, NEB #M0653T), 2 μL crRNA (100 nM), 2 μL 10× Cas12a Reaction Buffer, 1 μL FQ-reporter (10 μM), and variable RT-RAA product volumes, adjusted to 20 μL with DEPC-treated water. The reactions were incubated at 37 °C for 30 min in a QuantStudio 3 Real-Time PCR System (Applied Biosystems, Waltham, MA, USA) with fluorescence signals (FAM channel) recorded every 2 min.

### 2.6. Specificity Evaluation

The specificity of the RT-RAA-CRISPR/Cas12a assay was evaluated using genomic nucleic acids from porcine rotavirus (PoRV G3, G4), Transmissible Gastroenteritis Virus (TGEV, strain Purdue), Porcine Epidemic Diarrhea Virus (PEDV, strain CV777), porcine deltacoronavirus (PDCoV, strain HKU15), and 10 fecal samples from healthy pigs. Nucleic acids were extracted using the TIANamp Virus DNA/RNA Kit (TIANGEN, Beijing, China) for viral strains and the DNeasy Blood & Tissue Kit (Qiagen, Hilden, Germany) for fecal samples. Each pathogen (10^5^ copies/μL) and fecal nucleic acid (50 ng/μL) was tested under optimized conditions (Section 2.5 and Section 2.6), with DEPC-treated water as the negative control.

### 2.7. Sensitivity Assessment

The pUC57-PoRV-VP6 plasmid (1.25 × 10^8^ copies/μL, Sangon Biotech, Shanghai) was serially diluted (10-fold) to concentrations ranging from 1.25 × 10^3^ to 1.25 × 10^0^ copies/μL. Each dilution (1 μL) was tested in triplicate using the RT-RAA-CRISPR/Cas12a assay. Detection limits were compared against qPCR (SCT 7024-2021 protocol) using the 2^−ΔΔCt^ method.

### 2.8. Lateral Flow Dipstick (LFD) Validation

For visual detection, the FQ-reporter was replaced with a dual-labeled probe (5′-FAM-biotin-TTTTTTTATTTTTTT-3′, Sangon Biotech). Post-CRISPR/Cas12a reaction (30 μL), the products were diluted with 20 μL DEPC water and applied to HybriDetect LFDs (Milenia Biotec, Gießen, Germany). The results were interpreted after 10 min: the appearance of both test (T) and control (C) lines indicated target detection, while a single C-line denoted negativity.

### 2.9. Clinical Sample Validation and Statistical Analysis

Fifty diarrheic fecal samples from swine farms in Sanya, Hainan Province, and five spiked PoRV controls (10^3^ copies/μL) were analyzed. Nucleic acids were extracted as in Section 2.6 and tested via RT-RAA-CRISPR/Cas12a. The accuracy of clinical outcome detection was calculated using SPSS Statistics 26 software (IBM, Armonk, NY, USA).

## 3. Results

### 3.1. Primer Screening and Optimization

**Figure 1:**
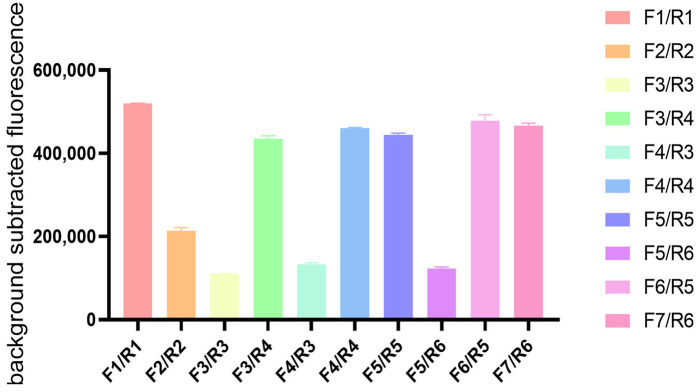
Screening of RPA primers for PoRV VP6 gene amplification. Agarose gel electrophoresis analysis of RT-RPA products generated with ten primer pairs (F1/R1 to F7/R6). Six primer combinations (F1/R1, F3/R4, F4/R4, F5/R5, F6/R5, F7/R6) demonstrated optimal amplification efficiency and were selected for subsequent experiments.

To establish an efficient RPA-CRISPR/Cas12a detection system for porcine rotavirus, ten primer pairs were initially evaluated for their amplification efficiency using RPA combined with agarose gel electrophoresis. As visualized in the electrophoretic profile (Figure 1), agarose gel electrophoresis analysis revealed that six primer pairs (F1/R1, F3/R4, F4/R4, F5/R5, F6/R5, and F7/R6) effectively amplified target sequences, indicating optimal amplification efficiency. Based on the evaluation criteria, including band clarity and product concentration, these six primer sets were selected for subsequent CRISPR system construction experiments.

### 3.2. Sensitivity Testing

**Figure 2:**
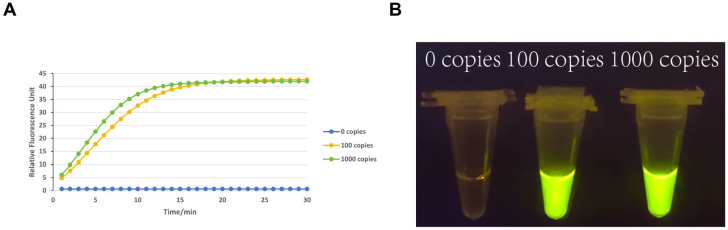
Sensitivity evaluation of the RPA-CRISPR/Cas12a detection system. (**A**) Kinetic fluorescence curves of serial dilutions (1.25 × 10^3^ to 1.25 × 10^0^ copies/μL) targeting PoRV VP6. (**B**) Visual fluorescence readouts under UV light, showing concentration-dependent signal intensity. The detection limit was determined as 10^2^ copies/μL.

To validate the detection sensitivity of the RPA-CRISPR/Cas12a system, serial dilutions of the porcine rotavirus target (1000 copies/μL and 100 copies/μL) were analyzed using the optimized F4/R4 primers and crRNA5. As shown in Figure 2A, the kinetic fluorescence curves demonstrated a clear dose-dependent response. For the 1000 copies/μL sample, fluorescence signals surged within 5 min (relative fluorescence unit [RFU] > 30) and plateaued at ~45 RFU by 30 min. At 100 copies/μL, a discernible signal increase (RFU > 15) was observed starting at 10 min, reaching ~35 RFU at 30 min. In contrast, the 0 copies/μL negative control exhibited negligible fluorescence (RFU < 5), confirming minimal background interference.

Visual readouts (Figure 2B) further corroborated the system’s sensitivity. Tubes containing 1000 copies/μL and 100 copies/μL targets displayed bright green fluorescence under UV light, with an intensity proportional to the target concentration. The 0 copies/μL tube remained non-fluorescent, maintaining a clear solution. These dual-signal outputs (quantitative fluorescence and qualitative visual brightness) aligned with the predefined sensitivity threshold of 100 copies/μL required for field applications. Given the stringent project specifications, we prioritized validating the 100 copies/μL detection limit. The consistent signal onset (<10 min) and robust fluorescence/visual differentiation at this concentration confirmed the assay’s capability to reliably identify low viral loads in resource-limited settings.

### 3.3. Screening of Amplification Components: Primer Selection and crRNA Evaluation

**Figure 3:**
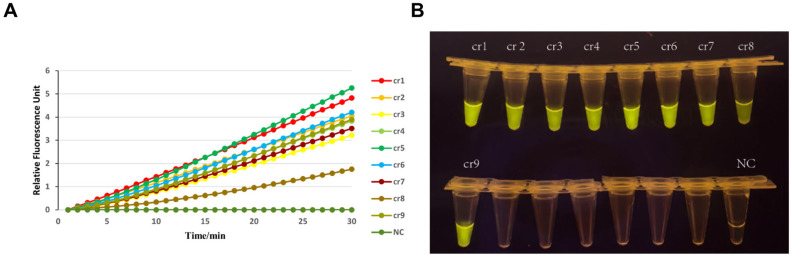
Screening of crRNAs for CRISPR/Cas12a activity. (**A**) Fluorescence kinetics of nine crRNAs (crRNA1–crRNA9) targeting conserved VP6 regions. (**B**) Visual colorimetric results (yellow precipitate formation) corresponding to crRNA5-mediated Cas12a trans-cleavage activity. crRNA5 exhibited the highest signal-to-noise ratio and was selected for subsequent assays.

Following the selection of optimal RPA primers, we systematically evaluated ten crRNAs (cr1–cr9 and NC) to identify the most effective guide RNA for 5CR0.CRISPR/Cas12a-mediated detection: As depicted in Figure 3, kinetic fluorescence curves (Figure 3A) revealed significant differences in Cas12a collateral cleavage activity across crRNAs. The crRNA5-driven reaction exhibited the highest RFU, with a rapid signal increase within 5 min and sustained amplification over 30 min (peak RFU: ~6 units), far exceeding other crRNAs (cr1–cr4, cr6–cr9: RFU ≤ 2; NC: negligible background). This stark contrast underscores crRNA5′s superior compatibility with the target amplicon (F4/R4 primer pair) and efficient activation of Cas12a trans-cleavage. Parallel visual readouts (Figure 3B) corroborated these findings: crRNA5-associated tubes displayed intense yellow precipitates at the reaction termini, indicative of robust nucleic acid degradation and pH-driven colorimetric shifts. In contrast, cr1–cr4, cr6–cr9, and NC tubes showed minimal or no color changes, aligning with their low fluorescence signals. The dual-readout consistency (quantitative fluorescence + qualitative visual signal) confirmed crRNA5′s exceptional specificity and efficiency in discriminating target sequences.

### 3.4. Sensitivity Confirmation and Optimization

**Figure 4:**
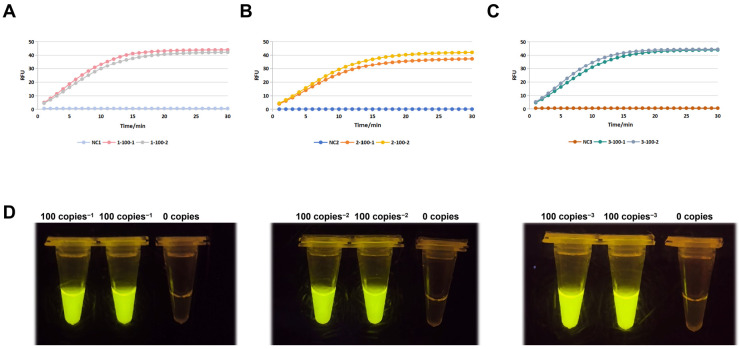
Reproducibility and validation of sensitivity thresholds. (**A**–**C**) Triplicate kinetic fluorescence profiles of 10^2^ copies/μL PoRV targets. (**D**) Gel imaging confirming consistent visual signals across biological replicates. The system achieved 100% detection accuracy at the 10^2^ copies/μL threshold.

To rigorously validate the optimized RPA-CRISPR/Cas12a system, triplicate experiments were conducted using 100 copies/μL porcine rotavirus targets (two technical replicates per run), with reaction conditions consistent with previous protocols. As shown in Figure 4A–C, the kinetic fluorescence profiles across all three independent runs (labeled Run 1–3) exhibited remarkable consistency. For 100 copies/μL targets, fluorescence signals initiated a steep ascent within 8–12 min, reaching peak RFU values of 42.3 ± 3.1 (Run 1), 40.8 ± 2.7 (Run 2), and 41.5 ± 2.9 (Run 3) by 30 min, significantly surpassing the background (NC: RFU < 5). The near-overlapping curves for technical replicates (e.g., Run 1: “1-100-1” vs. “1-100-2”) confirmed the minimal intra-experimental variability. The gel imaging results (Figure 4D) provided unambiguous visual confirmation. All three biological replicates (100 copies-1, -2, -3) displayed bright yellow fluorescence under UV excitation, with uniform signal intensity across replicates, while the 0 copies/μL controls remained non-fluorescent. This dual-readout concordance—quantitative RFU kinetics and qualitative visual positivity—eliminated ambiguity in interpreting low-concentration targets. Critically, the system achieved 100% detection accuracy (6/6 positive for 100 copies/μL; 0/3 false positives for NC) across all replicates, fulfilling the project’s sensitivity requirement of 100 copies/reaction. The rapid signal onset (<12 min) and robust inter-run reproducibility (CV < 8% for peak RFU) further underscore the assay’s suitability for field applications requiring rapid, low-resource pathogen detection. These results conclusively validate the optimized system’s sensitivity and reliability at the 100 copies/μL threshold, establishing a robust framework for point-of-care porcine rotavirus surveillance.

### 3.5. Specificity Testing

**Figure 5:**
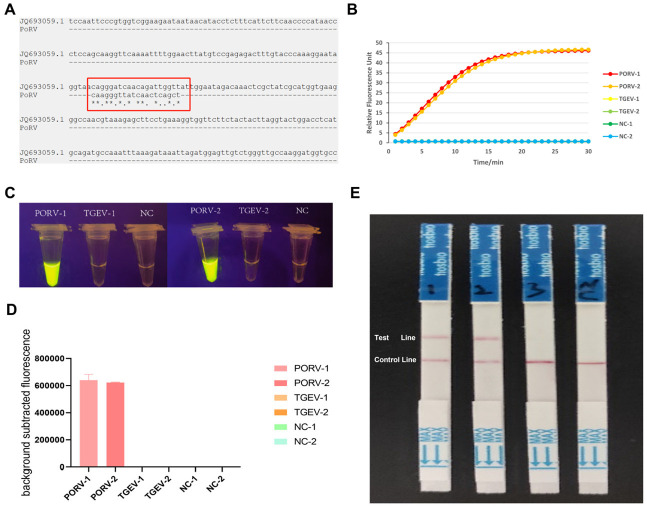
Specificity assessment of the RPA-CRISPR/Cas12a system. (**A**) MAFFT alignment of PoRV and TGEV sequences, highlighting 11/20 mismatches in crRNA binding regions. (**B**) Real-time fluorescence curves showing PoRV detection specificity. (**C**) Specificity test under UV light: Tubes from left to right: PoRV-1, TGEV-1, NC, PoRV-2, TGEV-2, and NC. Bright yellow fluorescence is exclusively observed in PoRV samples (arrows), while TGEV and negative control (NC) show no detectable signal. (**D**) Quantification of fluorescence signals: Bar chart of background-subtracted fluorescence values corresponding to (**C**). PoRV samples exhibit high fluorescence intensity (mean ± SD: 750,000 ± 32,000 RFU), whereas TGEV and NC groups show negligible values. (**E**) Lateral flow dipstick specificity validation for PoRV detection. Four dipsticks were tested (left to right): PoRV-1, PoRV-2, TGEV-1, and TGEV-2. Clear red bands at both the test and control lines were observed exclusively in PoRV-spiked samples, confirming positive detection. TGEV samples showed only control line bands, indicating no cross-reactivity. All control lines were visible, validating assay functionality.

The RPA-CRISPR/Cas12a system’s specificity was validated using TGEV, a porcine pathogen with clinical symptoms overlapping with PORV. Sequence alignment of the PORV-specific target sites was performed against the reference sequence using the MAFFT online service. As shown in the red-boxed region of Figure 5A, the alignment between the PORV Cas12a crRNA recognition sequence and the TGEV viral genome is presented, where asterisks denote identical nucleotides. Synthetic TGEV plasmids (400 bp flanking divergent regions) and PORV targets (100 copies/μL) were tested. Fluorescence curves (Figure 5B) showed strong PORV signals (RFU > 35) but negligible activity for TGEV and NC (RFU < 5), confirmed by UV-induced green fluorescence in the PORV samples only (Figure 5C,D). Lateral flow strips (Figure 5E) further validated the specificity: 10,000 and 1000 copies/μL PORV yielded clear test bands, while TGEV and NC showed only control bands. Dual-readout consistency and sequence divergence confirmed the system’s high specificity for PORV.

### 3.6. Clinical Sample Test Results

**Figure 6:**
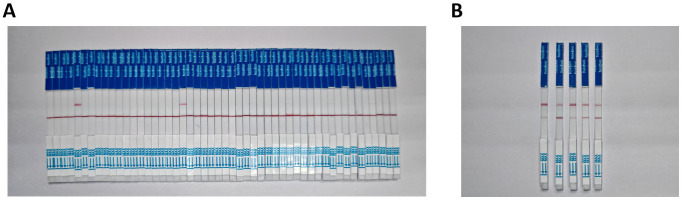
Detection performance of CRISPR/Cas12a-RPA assay for PoRV in clinical samples and the artificially contaminated samples. (**A**) CRISPR/Cas12a-RPA experiments detected clinical samples (2+/48−). (**B**) CRISPR/Cas12a-RPA experiments detected artificially contaminated samples (5+/0−).

The statistical results of the detection methods used in this study and the standard methods are shown in Table 3. The results in Table 3 demonstrate that the detection outcomes of the RT-RAA-CRISPR/Cas12a method developed in this study are completely consistent with those of the standard fluorescence method, with a concordance rate of 100%. This indicates that the RT-RAA-CRISPR/Cas12a method for detecting the PoRV is suitable for clinical testing and provides accurate and reliable results (Figure 6).

## 4. Discussion

The CRISPR/Cas12a dual-readout detection platform addresses critical gaps in current PoRV surveillance strategies [20,21,22]. By integrating recombinase polymerase amplification with CRISPR/Cas12a signal amplification, the platform achieves a sensitivity of 100 copies/μL within 1 h, representing a 10-fold improvement over conventional RT-PCR methods. Targeting the conserved VP6 gene and leveraging CRISPR’s collateral cleavage activity, the system demonstrates exceptional specificity, distinguishing PoRV from phylogenetically related viruses such as TGEV with 12/20 crRNA mismatch sites.

Three key innovations distinguish this platform [23]. First, the RPA-CRISPR/Cas12a integration overcomes the sensitivity limitations of standalone CRISPR systems while maintaining field applicability [24,25,26]. Second, the dual-readout design (fluorescence quantification and lateral flow visualization) accommodates diverse settings—enabling semi-quantitative analysis in laboratories and binary interpretations at farm sites. Third, a comprehensive crRNA screening strategy, informed by 200 VP6 sequences, ensures the detection of prevalent PoRV genotypes, critical given the virus’s high mutation rate in surface proteins. This approach contrasts with earlier CRISPR diagnostics, where off-target effects occasionally arose due to imperfect guide RNA specificity [27,28,29]. Our results emphasize the necessity of combining bioinformatic alignment tools with empirical crRNA screening to minimize false positives [30]. Compared to qPCR (10 copies/μL sensitivity), the platform sacrifices marginal sensitivity to gain field advantages: eliminating thermal cyclers (reducing equipment costs by ~80%), accelerating processing by 60% compared to nested PCR, and enabling visual interpretation without specialized training. These features position it as an ideal frontline screening tool, particularly in developing regions where 68% of swine farms lack molecular diagnostics. Furthermore, the system’s specificity was rigorously validated against TGEV and PDCoV, with no cross-reactivity observed—a result attributable to conserved VP6 targeting and optimized crRNA design. Despite these advancements, limitations warrant consideration [31]. The 100 copies/μL detection limit may miss early-stage infections with viral loads of <50 copies/μL, necessitating supplementary testing in subclinical cases. Additionally, while cross-reactivity with TGEV and PDCoV is excluded, broader validation against emerging recombinant strains is required to strengthen diagnostic confidence. Four discordant clinical samples (false negatives/positives) highlight the opportunities for optimization, potentially through periodic crRNA updates or enhanced primer specificity [31,32]. Future iterations could incorporate digital quantification hyperactive Cas12a variants, or nanomaterials to improve sensitivity for low-level viral shedding [33].

Notably, our CRISPR-Cas12a/RPA approach complements recent findings by Huang et al. [33]., who demonstrated that bacterial-derived sialidases inhibit PoRV OSU replication by targeting α-2,3-linked sialic acid receptors during early infection stages. While Huang et al. focused on blocking viral–host interactions (achieving 50% inhibition at 0.01 μU/mL sialidase), our method provides a field-deployable tool for rapid PoRV detection without requiring viral culture or receptor manipulation. The two strategies address distinct needs: Huang’s work proposes a novel antiviral mechanism targeting host receptors, whereas our assay enables on-site surveillance in resource-limited settings. Integrating these approaches could offer a synergistic solution—using sialidase pretreatment to reduce false negatives in low-viral-load samples while leveraging CRISPR-Cas12a for specificity.

The implications of this work extend beyond PoRV diagnostics [34]. The modular RPA-CRISPR/Cas12a framework could be adapted for other veterinary or zoonotic pathogens by redesigning primers and crRNAs, offering a versatile platform for emerging disease surveillance [35]. Future efforts should focus on multiplexing capabilities to address co-infections (e.g., with PEDV), integrating AI-driven crRNA design tools, and validating the system in large-scale, geographically diverse trials. Such advancements align with global efforts to democratize diagnostics, particularly in regions where swine farming underpins food security. By harmonizing speed, sensitivity, and simplicity, this platform exemplifies the transformative potential of CRISPR-based technologies in veterinary medicine, empowering timely interventions to safeguard swine health and economic stability [36,37].

## 5. Conclusions

This study presents a rapid, field-deployable RPA-CRISPR/Cas12a assay for porcine rotavirus detection, achieving a 10^2^ copies/μL sensitivity with dual visual/fluorescence readouts and no cross-reactivity, offering a cost-effective solution for on-site veterinary diagnostics.

## Figures and Tables

**Table 1:** The single primer sequences of the RPA primer design in this study, comprising 7 forward primers (F1–F7) and 6 reverse primers (R1–R6), are shown in the table.

Primer	Sequence (5′-3′)
F1	GTAACTTACCTGTTAGGAATTGGACTTTTG
F2	CCACAATCTGAAGCGTTAAGGAAGTTAGCAGG
F3	ACGCACCAGCGAATATACAACAGTTTGAGCAT
F4	GTTTGAGCATATTGTCCAACTTAGACGCGCAC
F5	CAATTCGCTTATCATTTCAATTAATGCGTCC
F6	TGCGTCCACCGAACATGACACCAGCTGTTAATGC
F7	GCTGTGCGTCAAGAGTATGCTATACCAGTTGG
R1	ACCTGCTAACTTCCTTAACGCTTCAGATTGTG
R2	CTGATCCAGCATTGAGCCACATAGTTCCCATC
R3	ATAACTGGGTTAAAGAACCATGTGGTTGCGCC
R4	GGTCTTAAAATAACTGGGTTAAAGAACCATGTGG
R5	ATACTCTTGACGCACAGCGGTCACATTCGCC
R6	AACACGTTGCAAATTATCCTCTCTGGATGGTG

**Table 2:** Based on the selected RPA primer combinations (combinations 1, 4, 6, 7, 9, and 10) with good amplification effects, crRNAs were designed within the amplified fragments of RPA.

crRNA	Sequence (5′-3′)
crRNA1	GGGUAAUUUCUACUAAGUGUAGAUGUCUAUUAGGCACAACACUC
crRNA2	GGGUAAUUUCUACUAAGUGUAGAUUCUGAGAUUCUCUCGCCAUU
crRNA3	GGGUAAUUUCUACUAAGUGUAGAUUGCAUCAGGCAACAAAGUUA
crRNA4	GGGUAAUUUCUACUAAGUGUAGAUUUGCCUGAUGCAGAAAGAUU
crRNA5	GGGUAAUUUCUACUAAGUGUAGAUCAAGGGUUAUCAACUCAGCU
crRNA6	GGGUAAUUUCUACUAAGUGUAGAUCGCAAGCACAACCCUCUCAA
crRNA7	GGGUAAUUUCUACUAAGUGUAGAUUGAAUCAGUGCUUGCGGAUG
crRNA8	GGGUAAUUUCUACUAAGUGUAGAUCAUCCGCAAGCACUGAUUCA
crRNA9	GGGUAAUUUCUACUAAGUGUAGAUCACCAGGCAUGAAUUGGACU

**Table 3:** The statistical results of the detection method used in this study and the standard method. The results in this table demonstrate complete concordance between the CRISPR/Cas12a-RPA assay and reference real-time RT-PCR across clinical (2+/48−) and artificially contaminated (5+/0−) samples, with both methods yielding identical detection outcomes for porcine rotavirus (PoRV) in all tested categories.

Sample Type	RT-RAA-CRISPR/Cas12a		Real-Time RT-PCR	
	Positive	Negative	Positive	Negative
Clinical sample	2	48	2	48
Artificially contaminated sample	5	0	5	0
Accuracy	100%		100%	

## Data Availability

All the data generated during the current study are included in the manuscript. Additional data related to this article may be requested from the corresponding authors.

## References

[1] Ghonaim A.H., Rouby S.R., Nageeb W.M., Elgendy A.A., Xu R., Jiang C., Ghonaim N.H., He Q., Li W. (2025). Insights into recent advancements in human and animal rotavirus vaccines: Exploring new frontiers. Virol. Sin..

[2] Huang Y., Zhu Q., Wang Y., Zhu K. (2024). Bacterial-derived sialidases inhibit porcine rotavirus OSU replication by interfering with the early steps of infection. Microb. Pathog..

[3] Chen Y., He Z., Luo Y., Su Q., Wang Q., Wang J., He J., Yu M., You H., Chen H. (2024). Tris stabilized AuNPs based lateral flow immunochromatography for the simultaneous detection of porcine epidemic diarrhea virus and rotavirus on-site. Spectrochim. Acta A Mol. Biomol. Spectrosc..

[4] Yin Y., Zhu L., Liu P., Zhao J., Fan Y., Sun X., Xu Z. (2019). Evaluation on the efficacy and immunogenicity of recombinant DNA plasmids expressing S gene from porcine epidemic diarrhea virus and VP7 gene from porcine rotavirus. Braz. J. Microbiol..

[5] Jia S., Feng B., Wang Z., Ma Y., Gao X., Jiang Y., Cui W., Qiao X., Tang L., Li Y. (2019). Dual priming oligonucleotide (DPO)-based real-time RT-PCR assay for accurate differentiation of four major viruses causing porcine viral diarrhea. Mol. Cell. Probes.

[6] De La Cruz Hernandez S.I., Anaya Molina Y., Gomez Santiago F., Teran Vega H.L., Monroy Leyva E., Mendez Perez H., Garcia Lozano H. (2018). Real-time RT-PCR, a necessary tool to support the diagnosis and surveillance of rotavirus in Mexico. Diagn. Microbiol. Infect. Dis..

[7] Li H., Bello A., Smith G., Kielich D.M.S., Strong J.E., Pickering B.S. (2022). Degenerate sequence-based CRISPR diagnostic for Crimean-Congo hemorrhagic fever virus. PLoS Negl. Trop. Dis..

[8] Han W., Ma Z., Li Z., Chang C., Yuan Y., Li Y., Feng R., Zheng C., Shi Z., Tian H. (2024). A novel double antibody sandwich quantitative ELISA for detecting porcine epidemic diarrhea virus infection. Appl. Microbiol. Biotechnol..

[9] Huang S., Du L., Liu S., Yang Q., Lei C., Wang H., Yang L., Yang X. (2024). Development and Validation of RAA-CRISPR/Cas12a-Based Assay for Detecting Porcine Rotavirus. Animals.

[10] Shi K., Zhou H., Feng S., He J., Li B., Long F., Shi Y., Yin Y., Li Z. (2023). Development of a Quadruplex RT-qPCR for the Detection of Porcine Rotaviruses and the Phylogenetic Analysis of Porcine RVH in China. Pathogens.

[11] Pan Y., Li Z., Miao Q., Shi H., Guo L., Feng L., Tian J. (2025). Phylogenitc analysis and immunogenicity comparison of porcine genotype G9 rotavirus in China from 2020–2023. Virol. Sin..

[12] Almeida P.R., Lorenzetti E., Cruz R.S., Watanabe T.T., Zlotowski P., Alfieri A.A., Driemeier D. (2018). Diarrhea caused by rotavirus A, B, and C in suckling piglets from southern Brazil: Molecular detection and histologic and immunohistochemical characterization. J. Vet. Diagn. Investig..

[13] Shahni S.N., Albogami S., Azmi I., Pattnaik B., Chaudhuri R., Dev K., Iqbal J., Sharma A., Ahmad T. (2024). Dual Detection of Hepatitis B and C Viruses Using CRISPR-Cas Systems and Lateral Flow Assay. J. Nucleic Acids.

[14] Brogan D.J., Akbari O.S. (2023). CRISPR Diagnostics: Advances toward the Point of Care. Biochemistry.

[15] Garrison A.R., Moresco V., Zeng X., Cline C.R., Ward M.D., Ricks K.M., Olschner S.P., Cazares L.H., Karaaslan E., Fitzpatrick C.J. (2024). Nucleocapsid protein-specific monoclonal antibodies protect mice against Crimean-Congo hemorrhagic fever virus. Nat. Commun..

[16] Shariq M., Khan M.F., Raj R., Ahsan N., Singh R., Kumar P. (2023). CRISPR-based diagnostic approaches: Implications for rapid management of future pandemics (Review). Mol. Med. Rep..

[17] Zhou L., Simonian A.L. (2024). CRISPR/Cas Technology: The Unique Synthetic Biology Genome-Editing Tool Shifting the Paradigm in Viral Diagnostics, Defense, and Therapeutics. Annu. Rev. Biomed. Eng..

[18] Luo T., Li K., Li C., Xia C., Gao C. (2024). Development of a triplex quantitative reverse transcription-polymerase chain reaction for the detection of porcine epidemic diarrhea virus, porcine transmissible gastroenteritis virus, and porcine rotavirus A. Front. Microbiol..

[19] Kostyusheva A., Brezgin S., Babin Y., Vasilyeva I., Glebe D., Kostyushev D., Chulanov V. (2022). CRISPR-Cas systems for diagnosing infectious diseases. Methods.

[20] Qu H., Zhang W., Li J., Fu Q., Li X., Wang M., Fu G., Cui J. (2024). A rapid and sensitive CRISPR-Cas12a for the detection of *Fusobacterium nucleatum*. Microbiol. Spectr..

[21] Liu Q., Zeng H., Wang T., Ni H., Li Y., Qian W., Fang T., Xu G. (2024). Development of RPA-Cas12a assay for rapid and sensitive detection of *Pneumocystis jirovecii*. BMC Microbiol..

[22] Kim H.J., Cho I.S., Choi S.R., Jeong R.D. (2024). Identification of an Isolate of Citrus Tristeza Virus by Nanopore Sequencing in Korea and Development of a CRISPR/Cas12a-Based Assay for Rapid Visual Detection of the Virus. Phytopathology.

[23] Cao X., Chang Y., Tao C., Chen S., Lin Q., Ling C., Huang S., Zhang H. (2023). Cas12a/Guide RNA-Based Platforms for Rapidly and Accurately Identifying *Staphylococcus aureus* and Methicillin-Resistant *S. aureus*. Microbiol. Spectr..

[24] Jiao J., Kong K., Han J., Song S., Bai T., Song C., Wang M., Yan Z., Zhang H., Zhang R. (2021). Field detection of multiple RNA viruses/viroids in apple using a CRISPR/Cas12a-based visual assay. Plant Biotechnol. J..

[25] Ling C., Chang Y., Wang X., Cao X., Tu Q., Liu B., Huang S. (2023). Two CRISPR/Cas12a-based methods for fast and accurate detection of single-base mutations. Anal. Chim. Acta.

[26] Huang S., Liu Y., Zhang X., Gai Z., Lei H., Shen X. (2023). A Rapid RPA-CRISPR/Cas12a Detection Method for Adulteration of Goat Milk Powder. Foods.

[27] Yan J., Xu Z., Zhou H., Li T., Du X., Hu R., Zhu J., Ou G., Li Y., Yang Y. (2022). Integration of CRISPR/Cas12a and Multiplexed RPA for Fast Detection of Gene Doping. Anal. Chem..

[28] Wang P., Guo B., Zhang X., Wang Y., Yang G., Shen H., Gao S., Zhang L. (2023). One-Pot Molecular Diagnosis of Acute Hepatopancreatic Necrosis Disease by Recombinase Polymerase Amplification and CRISPR/Cas12a with Specially Designed crRNA. J. Agric. Food Chem..

[29] Zeng L., Zheng S., Stejskal V., Opit G., Aulicky R., Li Z. (2023). New and rapid visual detection assay for *Trogoderma granarium* everts based on recombinase polymerase amplification and CRISPR/Cas12a. Pest Manag. Sci..

[30] Chen Y., Xu X., Wang J., Zhang Y., Zeng W., Liu Y., Zhang X. (2022). Photoactivatable CRISPR/Cas12a Strategy for One-Pot DETECTR Molecular Diagnosis. Anal. Chem..

[31] Lin K., Guo J., Guo X., Li Q., Li X., Sun Z., Zhao Z., Weng J., Wu J., Zhang R. (2023). Fast and visual detection of nucleic acids using a one-step RPA-CRISPR detection (ORCD) system unrestricted by the PAM. Anal. Chim. Acta.

[32] Li X., Liu M., Men D., Duan Y., Deng L., Zhou S., Hou J., Hou C., Huo D. (2024). Rapid, portable, and sensitive detection of CaMV35S by RPA-CRISPR/Cas12a-G4 colorimetric assays with high accuracy deep learning object recognition and classification. Talanta.

[33] He Q., Yu D., Bao M., Korensky G., Chen J., Shin M., Kim J., Park M., Qin P., Du K. (2020). High-throughput and all-solution phase African Swine Fever Virus (ASFV) detection using CRISPR-Cas12a and fluorescence based point-of-care system. Biosens. Bioelectron..

[34] Zhang H., Li Z., Daczkowski C.M., Gabel C., Mesecar A.D., Chang L. (2019). Structural Basis for the Inhibition of CRISPR-Cas12a by Anti-CRISPR Proteins. Cell Host Microbe.

[35] Wang X., Chen X., Xu T., Jin X., Jiang J., Guan F. (2024). Rapid and Ultrasensitive Detection of H. aduncum via the RPA-CRISPR/Cas12a Platform. Molecules.

[36] Hu M., Qiu Z., Bi Z., Tian T., Jiang Y., Zhou X. (2022). Photocontrolled crRNA activation enables robust CRISPR-Cas12a diagnostics. Proc. Natl. Acad. Sci. USA.

[37] MacGregor S.R., McManus D.P., Sivakumaran H., Egwang T.G., Adriko M., Cai P., Gordon C.A., Duke M.G., French J.D., Collinson N. (2023). Development of CRISPR/Cas13a-based assays for the diagnosis of Schistosomiasis. EBioMedicine.

